# Probiotics and microbiota composition

**DOI:** 10.1186/s12916-016-0629-z

**Published:** 2016-06-02

**Authors:** Mary Ellen Sanders

**Affiliations:** Dairy & Food Culture Technologies, 7119 S. Glencoe Ct., Centennial, CO 80122 USA

**Keywords:** Probiotic, *Lactobacillus*, Homeostasis, Gut microbiota, Metagenomics

## Abstract

Accumulated evidence, corroborated by a new systematic review by Kristensen et al. (Genome Med 8:52, 2016), suggests that probiotics do not significantly impact the fecal microbiota composition of healthy subjects. Nevertheless, physiological benefits have been associated with probiotic consumption by healthy people. Some studies have suggested that probiotics may impact the function of colonizing microbes, although this needs to be further studied. An alternative hypothesis is that probiotics may promote homeostasis of the gut microbiota, rather than change its composition. This hypothesis warrants investigation as a possible mechanism for how probiotics may benefit healthy people.

Please see related article: http://genomemedicine.biomedcentral.com/articles/10.1186/s13073-016-0300-5.

## Background

The consumption of probiotics has been reported to induce a range of benefits for human health, including the prevention of necrotizing enterocolitis in premature infants [1], crying time reduction in colicky babies [2], reduction in acute pediatric diarrhea duration [3], symptom management in irritable bowel syndrome [4], and prevention of antibiotic-associated diarrhea [5]. The underlying mechanism for probiotic functionality is often assumed to stem from their ability to impact the human microbiota. However, in a study recently published by Kristensen et al. [6], the conclusion is that the probiotics tested thus far do not have a substantive effect on the overall composition of colonizing gut microbes in healthy adults. Nevertheless, this finding should not be interpreted to mean that probiotics have no effect on healthy adults; indeed, numerous controlled intervention trials argue otherwise. However, Kristensen et al.’s [6] study does suggest that an alteration in gut microbiota composition is not a primary mechanism of probiotic functionality.

The extent of the evidence considered in Kristensen et al.’s [6] review comprises seven randomized controlled trials that assessed fecal samples from healthy subjects using shotgun metagenomic sequencing, 16S rRNA sequencing, or phylogenetic microarray methods. While most of the included studies covered *Lactobacillus* probiotics, the review also included one study each for *Bifidobacterium longum* or *Bacillus subtilis*. Using a systematic approach, the authors found no effect of probiotics on fecal microbiota composition when compared to a placebo, as reflected by alpha-diversity, richness, or evenness. Not included in the Kristensen et al. [6] study were pre-metagenomic studies, which have demonstrated that probiotic consumption often increases the number of related phylotypes and, in some cases, decreases opportunistic pathogens and their toxins [7]. Such limited effects are likely masked in comprehensive metagenomics assessments. These limited compositional changes notwithstanding, Kristensen et al.’s [6] review challenges us to reconsider assumptions regarding the mechanisms behind the documented efficacy of probiotics.

### Probiotic impact on the microbiota

Although outside the scope of the review by Kristensen et al. [6], another aspect that should be considered is whether or not probiotics exert an effect on the function of microbiota as reflected by metatranscriptomic and metabolomic analyses. Indeed, Eloe-Fadrosh et al. [8] and McNulty et al. [9] have described such effects. However, relevance to human health of the metabolic changes observed thus far remains to be elucidated. The same can be said for compositional changes. We do not know how colonizing populations are established or the causes of their variability over time. At any given moment, gut microbiota composition is impacted by so many host and environmental variables that it is difficult to form meaningful hypotheses. This point is clearly made through a recent microbiome analysis of fecal samples from a total of 3948 healthy subjects, tracking just over 500 metadata variables [10]. Sixty-nine factors were shown to correlate significantly with overall microbiome community variation reflected in alpha-diversity and abundances, yet these variables explained only a small fraction of the variation of genera present in the communities. Even this large study was not able to determine the essential factors responsible for determining the composition of our gut microbiota.

**Fig. 1:**
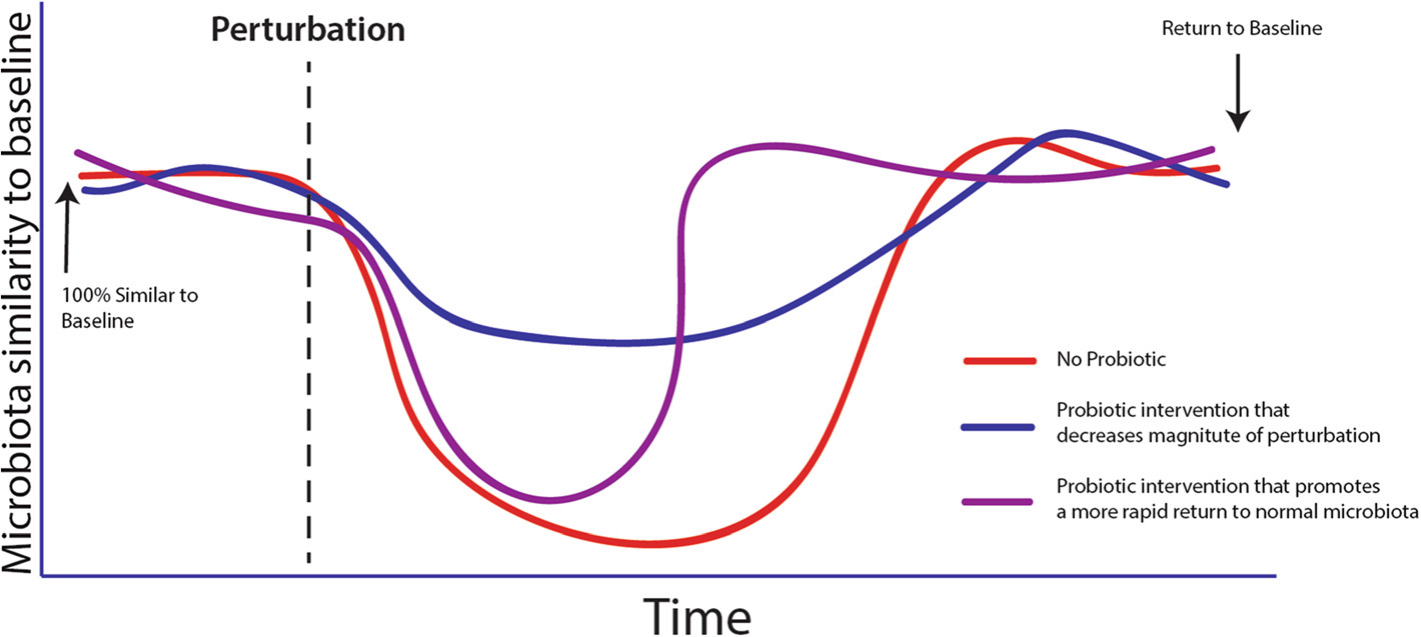
The concept of homeostasis as expressed by reducing the magnitude or duration of the impact of a stress on the microbiota. Modified from Sanders et al. [12], no permission required

A pressing topic in the probiotic field today is whether or not probiotics can impact gut microbiota in a manner that improves the health of the host. Unfortunately, since the composition of a healthy microbiota remains unknown [11], there is a lack of robust phylogenetic targets for exploratory research. Rather than focusing on specific phylogenetic changes in composition, a more fruitful approach could be to assess the ability of probiotics to promote microbiota stability [12]. Although not a new concept, surprisingly few studies have addressed the ability of a probiotic to reinforce the colonizing microbiota’s ability to either resist perturbation to stressors (for example, antibiotics, poor diet, psychological stress) or quicken recovery from said stress. Engelbreckston et al. [13] showed less antibiotic-induced microbiota disruption in healthy, probiotic-supplemented adults than in those who did not take a probiotic. Their study assessed microbiota changes using both culture techniques and terminal restriction fragment length polymorphism. Studies comparing metagenomic composition before and after a stress, with and without a probiotic intervention, could provide insights into the ability of probiotics to support host health through stabilizing the microbiota, rather than fundamentally changing its composition (Fig. 1).

## Conclusions

Kristensen et al. [6] have provided incentives to revise our assumptions of how probiotics might promote health in humans. Additional research is needed to clarify whether probiotics can instead promote gut microbiota homeostasis and thereby minimize the far reaching effects of microbiota disturbances. Such research may help resolve this apparent contradiction between the clear health benefits of probiotics and their lack of impact on microbiota composition.

## References

[1] Lau CS, Chamberlain RS (2015). Probiotic administration can prevent necrotizing enterocolitis in preterm infants: a meta-analysis. J Pediatr Surg.

[2] Harb T, Matsuyama M, David M, Hill RJ (2016). Infant colic-what works: a systematic review of interventions for breast-fed infants. J Pediatr Gastroenterol Nutr.

[3] Szajewska H, Skorka A, Ruszczynski M, Gieruszczak-Bialek D (2013). Meta-analysis: Lactobacillus GG for treating acute gastroenteritis in children--updated analysis of randomised controlled trials. Aliment Pharmacol Ther.

[4] Didari T, Mozaffari S, Nikfar S, Abdollahi M (2015). Effectiveness of probiotics in irritable bowel syndrome: updated systematic review with meta-analysis. World J Gastroenterol.

[5] Goldenberg JZ, Lytvyn L, Steurich J, Parkin P, Mahant S, Johnston BC (2015). Probiotics for the prevention of pediatric antibiotic-associated diarrhea. Cochrane Database Syst Rev.

[6] Kristensen NB, Bryrup T, Allin KH, Nielsen T, Hansen TH, Pedersen O (2016). Alterations in fecal microbiota composition by probiotic supplementation in healthy adults: a systematic review of randomized controlled trials. Genome Med.

[7] Sanders ME (2011). Impact of probiotics on colonizing microbiota of the gut. J Clin Gastroenterol.

[8] Eloe-Fadrosh EA, Brady A, Crabtree J, Drabek EF, Ma B, Mahurkar A, Ravel J, Haverkamp M, Fiorino AM, Botelho C (2015). Functional dynamics of the gut microbiome in elderly people during probiotic consumption. MBio.

[9] McNulty NP, Yatsunenko T, Hsiao A, Faith JJ, Muegge BD, Goodman AL, Henrissat B, Oozeer R, Cools-Portier S, Gobert G (2011). The impact of a consortium of fermented milk strains on the gut microbiome of gnotobiotic mice and monozygotic twins. Sci Transl Med.

[10] Falony G, Joossens M, Vieira-Silva S, Wang J, Darzi Y, Faust K, Kurilshikov A, Bonder MJ, Valles-Colomer M, Vandeputte D (2016). Population-level analysis of gut microbiome variation. Science.

[11] Backhed F, Fraser CM, Ringel Y, Sanders ME, Sartor RB, Sherman PM, Versalovic J, Young V, Finlay BB (2012). Defining a healthy human gut microbiome: current concepts, future directions, and clinical applications. Cell Host Microbe.

[12] Sanders ME, Heimbach JT, Pot B, Tancredi DJ, Lenoir-Wijnkoop I, Lahteenmaki-Uutela A, Gueimonde M, Banares S (2011). Health claims substantiation for probiotic and prebiotic products. Gut Microbes.

[13] Engelbrektson A, Korzenik JR, Pittler A, Sanders ME, Klaenhammer TR, Leyer G, Kitts CL (2009). Probiotics to minimize the disruption of faecal microbiota in healthy subjects undergoing antibiotic therapy. J Med Microbiol.

